# Effectiveness of Educational Programmes on Knowledge and Practice Related to Diabetic Foot Care Among Nurses: A Systematic Review

**DOI:** 10.1002/edm2.70324

**Published:** 2026-08-27

**Authors:** Mohammad Al‐Me'ani, Omar Alqaisi, Mohammed Dibas, Heyam Al‐Aaraj, Yousef Rubbai

**Affiliations:** ^1^ Clinical Nursing Department, Faculty of Nursing Middle East University Amman Jordan; ^2^ Nursing Department Al‐Zaytoonah University Amman Jordan; ^3^ Department of Medicine, Faculty of Medicine and Health Sciences An‐Najah National University Nablus Palestine; ^4^ Community Health Nursing Department, Faculty of Nursing Middle East University Amman Jordan; ^5^ Princess Aisha Bint Al‐Hussein College of Nursing Ma'an Jordan

**Keywords:** diabetic foot care, diabetic foot ulcer, educational intervention, educational programmes, knowledge, nurses, practice, training programmes

## Abstract

**Background:**

Diabetes mellitus is a leading global non‐communicable disease associated with significant morbidity, mortality and healthcare burden. Diabetic foot complications represent a leading cause of hospitalization and lower‐limb amputation in this population.

**Objective:**

To systematically evaluate the effects of educational programmes on nurses' knowledge and practice regarding diabetic foot care.

**Methods:**

A systematic search of quasi‐experimental studies from 2015 to 2024 was performed. Web of Science, Cochrane Library, CINAHL, MEDLINE and Scopus databases were searched using a comprehensive search strategy. The Joanna Briggs Institute Critical Appraisal Checklist for Quasi‐Experimental Studies was used to evaluate methodological quality. The results were summarized narratively due to variations in study designs, interventions and outcome measures. The GRADE approach was used for assessment of the evidence certainty.

**Results:**

A total of nine studies met the inclusion criteria. Educational interventions were found to be consistently linked to enhanced knowledge and/or clinical practice concerning diabetic foot care among nurses in the included studies. Interactive and practical educational approaches appeared particularly promising; however, considerable heterogeneity existed in intervention content, duration, measurement instruments and follow‐up periods. The overall certainty of evidence for both knowledge and practice outcomes was rated as very low.

**Conclusion:**

Educational programmes were consistently associated with improvements in nurses' knowledge and practice regarding diabetic foot care. The findings suggest that healthcare training could be integrated with ongoing professional development programmes, emphasizing experiential learning. However, the very low certainty of evidence and predominance of non‐randomized quasi‐experimental designs limit causal inference. More rigorous randomized or cluster‐randomized studies with standardized outcome measures, longer follow‐up and patient‐level outcomes are needed.

**Trial Registration:**

PROSPERO: CRD420261404535

## Graphical abstract

Educational programmes consistently improved nurses' knowledge and/or practice in diabetic foot care across nine quasi‐experimental studies. However, the certainty of evidence was very low because of methodological limitations and heterogeneity, highlighting the need for high‐quality randomized studies to confirm effectiveness and long‐term outcomes.

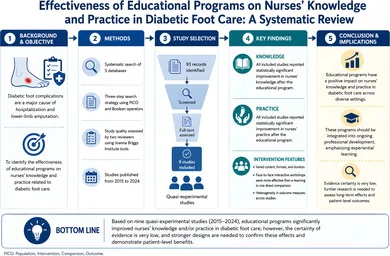

## 1 Introduction

Diabetes is one of the most common chronic non‐communicable diseases globally, and it is associated with high rates of morbidity, mortality and high healthcare costs [1]. Recent estimates indicate that 537 million adults were living with diabetes in 2021, and this number is projected to rise to 783 million by 2045, with the largest relative increase expected in low‐ and middle‐income countries [1]. In many regions, including the Middle East, the burden of diabetes is increasing rapidly as a result of lifestyle changes, urbanization and population aging [2, 3].

Among the chronic complications of diabetes, diabetic foot is one of the most serious, as it is a major cause of infection, hospitalization and lower limb amputation [4, 5]. International data indicate that between 19% and 34% of people with diabetes will develop a diabetic foot ulcer during their lifetime, and that the majority of lower limb amputations not caused by injuries are preceded by a foot ulcer [4, 5].

The International Working Group on Diabetic Foot (IWGDF) defines diabetic foot as any infection, ulceration or damage to the foot that has been confirmed or has a history of diabetes and must also have peripheral morbidity and/or diabetic peripheral disease [5]. This includes a loss of protective sensation, foot deformities and impaired blood flow, which greatly increase the risk of ulceration and delay healing [6]. Despite its serious consequences, diabetic foot can be largely prevented through systematic, evidence‐based interventions.

International guidelines emphasize the importance of foot examination, early detection of at‐risk patients, appropriate footwear selection, prompt treatment of minor injuries and structured patient health education as essential elements of prevention [2, 5, 6]. Systematic screening and timely multidisciplinary management have been shown to reduce the incidence of foot ulcers and amputations and improve the quality of life of people with diabetes [4, 6].

Nurses play a crucial role in the early detection and prevention of diabetic foot problems at all stages of care. In many hospital departments, outpatient diabetes clinics, primary healthcare centres and community or home care services, nurses are among the health professionals who have frequent and continuous contact with patients [6, 7]. Their responsibilities often include conducting routine foot assessments, identifying early signs of neuropathy and peripheral artery disease, educating patients about foot self‐care and coordinating timely referrals to specialist foot services [2, 7]. However, studies from various countries have documented significant gaps in nurses' knowledge and practice in assessing diabetic foot and in applying evidence‐based preventive measures, as well as limited access to structured preparation in this area [8, 9, 10].

To address these gaps, several educational programmes and training initiatives have been developed to improve nurses' competencies in diabetic foot care. International and national guidelines recommend that healthcare professionals involved in diabetes care receive regular competency‐based education on risk assessment, standard foot examination, use of simple diagnostic tools such as 10‐g monofilaments and tuning forks, shoe evaluation and patient counselling [5, 6, 7]. Nevertheless, the available evidence on the effectiveness of such educational interventions for nurses is scattered across different settings and study designs, and there is no clear consensus regarding the most effective format, intensity or content of these programmes [8, 9].

Therefore, a systematic review of quasi‐experimental studies evaluating educational programmes for nurses in the context of diabetic foot care is warranted. The present review aims to identify and critically appraise quasi‐experimental studies published between 2015 and 2024 that examined the effectiveness of educational programmes on nurses' knowledge and practice in diabetic foot care. By summarizing the characteristics and outcomes of these interventions, this review seeks to inform educators, clinical leaders and policymakers about strategies that may enhance nurses' competencies and ultimately improve the prevention and management of diabetes‐related foot complications.

## 2 Methods

### 2.1 Protocol and Registration

The protocol for this systematic review was prospectively registered in the International Prospective Register of Systematic Reviews (PROSPERO) [11].

### 2.2 Search Strategy

The development and reporting of the search strategy were guided by the PRISMA 2020 statement for systematic reviews [12]. An initial limited search of Web of Science, Cochrane Library, EBSCOhost (CINAHL), MEDLINE and Scopus was undertaken followed by analysis of the keywords and index terms contained in the title and abstract. A second‐step search using all identified keywords and index terms was then undertaken across all included databases. Then the reference list of all identified reports and articles was searched for additional studies. Studies published in non‐English languages were not considered for inclusion in this review, due to lack of available resources for translation. Studies published from 2015 to 2024 were considered for inclusion in this review. Initial keywords used were: ‘Educational Programme’; ‘Educational Intervention’; ‘Training programme’; ‘Diabetic Foot Care’; ‘diabetic foot ulcer’; ‘Knowledge’; ‘Practice’; ‘Nurses’. All databases were last searched on 15 January 2025. The full search strategies for all databases, including Boolean operators, field tags and applied filters, are provided in Appendix S1.

### 2.3 PICO Model

This review was performed according to the PICO framework (Population, Intervention, Comparison and Outcome) using Boolean operators (AND, OR) to completely retrieve studies that may have used alternative words and/or combinations (Table 1).

**TABLE 1:** PICO framework used to formulate the review question and eligibility criteria.

Population	Nurses
Intervention	Educational programme toward diabetic foot care
Comparison	Pre‐intervention (baseline) levels of knowledge and practice compared with post‐intervention levels
Outcome	Improvement in levels of knowledge and practice among nurses related to diabetic foot care

### 2.4 PICO Question

In nurses (P), how does an educational programme about diabetic foot care (I), compared with baseline or usual care (C), affect levels of knowledge and practice regarding diabetic foot care (O)?

### 2.5 Eligibility Criteria

Studies were either included or excluded based on criteria built up and identified from the aims of the study and the initial search of the literature. Studies were selected based on the title and abstract and were searched, selected and analyzed not only for their eligibility but also for quality in terms of type of studies, type of participants, type of interventions and outcome measures. In most of the included quasi‐experimental studies, the comparison was between nurses' pre‐intervention baseline and post‐intervention levels of knowledge and practice rather than between randomized intervention and control groups.

### 2.6 Inclusion Criteria

- Studies evaluating educational programmes related to diabetic foot care among nurses.
- Quasi‐experimental study design.
- Studies published from 2015 to 2024.
- Full‐text articles.

### 2.7 Exclusion Criteria

- Articles evaluating educational programmes among patients.
- Studies involving mixed interventions.
- Studies with a JBI critical appraisal score of 5 or less out of nine items.
- Non‐English language publications.

### 2.8 Quality Assessment

The studies selected for retrieval were assessed by two independent authors (M.A., O.A.) for methodological validity prior to inclusion in the review using the Joanna Briggs Institute (JBI) standardized critical appraisal checklist for quasi‐experimental studies [13]. Any disagreements between authors were resolved through discussion and consensus, or by consultation with a third author (M.D.) when necessary. The JBI critical appraisal checklist for quasi‐experimental studies comprises nine items evaluating the internal validity of non‐randomized experimental studies across key domains, including clarity of causal relationships, comparability of groups, similarity of treatment, measurement of outcomes before and after the intervention, completeness of follow‐up, consistency of outcome measurement, reliability of outcome measures and appropriateness of statistical analysis [13]. The methodological quality assessment of the included studies is presented in Table 2.

**TABLE 2:** Methodological quality assessment of the included studies using the Joanna Briggs Institute (JBI) Critical Appraisal Checklist for Quasi‐Experimental Studies.

No.	Study title	Author/Reference	Study design	JBI score	Inclusion decision
1	Effect of educational program about foot care on nurses' knowledge, practice and outcomes for patients with diabetes	Waheida et al. [14]	Quasi‐experimental pre‐posttest control trial	6/9	Included
2	Diabetic foot workshop: Improving technical and educational skills for nurses	Aalaa et al. [15]	Quasi‐experimental study	6/9	Included
3	Knowledge of diabetic foot care among nursing practitioners after interventional training in Rivers State, Nigeria	Buloala and John [16]	Quasi‐experimental study	6/9	Included
4	Effect of interventional education on the practice of special diabetic foot care among nurses in Rivers State	Buloala [17]	Quasi‐experimental study	6/9	Included
5	Intervention study of a foot‐care programme enhancing knowledge and practice among nurses and care workers at in‐home service providers	Fujii and Stolt [18]	Non‐randomized controlled study with cluster sampling	7/9	Included
6	Diabetic foot care course: A quasi‐experimental study on e‐learning versus interactive workshop	Aalaa et al. [19]	Quasi‐experimental two‐group pre‐post study	7/9	Included
7	Knowledge of primary care nurses before and after educational intervention on diabetic foot	Felix et al. [20]	Quasi‐experimental before‐and‐after study	7/9	Included
8	Effectiveness of educational program on knowledge and practices of nurses regarding prevention of diabetic foot ulcers at tertiary care hospital, Lahore	Ramzan et al. [21]	Quasi‐experimental pre‐post study	6/9	Included
9	Effect of foot care training program on knowledge and practices of nurses caring for diabetic geriatric patients	AbdElshafy et al. [22]	Quasi‐experimental pre‐post study	6/9	Included

*Note:* Studies scoring 6/9 or above were considered eligible for inclusion in the review.

### 2.9 Certainty of Evidence (GRADE)

Alongside the methodological quality appraisal using the JBI checklist, we assessed the overall certainty of evidence for the primary outcomes utilizing the Grading of Recommendations Assessment, Development and Evaluation (GRADE) approach. Given the non‐randomized, quasi‐experimental design of all included studies, the initial certainty of evidence was designated as ‘low’ for each outcome. We subsequently evaluated the evidence for potential downgrading across five core GRADE domains: risk of bias, inconsistency, indirectness, imprecision and publication bias.

Risk of bias was judged as serious due to the lack of randomization and, in most instances, the absence of concurrent control groups. Inconsistency was also deemed serious because of substantial heterogeneity in intervention content, delivery duration, measurement instruments and follow‐up periods. Similarly, imprecision was classified as a serious concern, as several studies featured relatively small sample sizes and lacked long‐term outcome data. Conversely, indirectness was not considered serious, because the target population (nurses), interventions (educational programmes) and measured outcomes (knowledge and clinical practice regarding diabetic foot care) directly aligned with the review's objective. Finally, while publication bias could not be formally calculated, it was considered a potential risk given the small number of included studies and the predominance of positive findings.

Two reviewers independently applied the GRADE criteria to the body of evidence for each outcome, with any discrepancies resolved through discussion to reach a consensus. The final certainty ratings and a Summary of Findings table are detailed in the Results section.

### 2.10 Data Extraction

Data were extracted from the included studies using a standardized data extraction form developed for this review. The extracted data included study characteristics, population and sample size, eligibility criteria, study design, study objectives, intervention characteristics, comparator, outcome measures and findings relevant to the review question. Data on nurses' knowledge and practice outcomes, including pre‐ and post‐intervention scores, proportions and corresponding *p*‐values where reported, were also extracted. The extracted study characteristics and main findings are summarized in Table 3. Included studies were classified as Level II evidence according to the Johns Hopkins Evidence‐Based Practice Model [23].

**TABLE 3:** Study characteristics and main findings of the included studies.

Author, year	Aim of study	Sample size	Intervention; study comparator	Main findings
Waheida et al. 2015 [14]	To determine the effect of an educational programme about foot care on nurses' knowledge, practice and patient outcomes	30 nurses working in the medical department at Tanta University Hospital and 40 adult diabetic patients without previous foot ulcer	Structured nursing educational programme on diabetic foot care using theoretical and practical sessions, plus implementation of a 60‐s diabetic foot screening test. Comparator: pre‐intervention baseline knowledge and practice scores	Nurses' knowledge and practice scores improved significantly immediately and 1 month after the programme (*p* < 0.001). Good total practical scores reached 93.33% immediately and 90% at 1‐month follow‐up. Patient knowledge and self‐care practices also improved significantly (*p* < 0.001)
Aalaa et al. 2017 [15]	To improve nurses' technical and educational skills for prevention and management of diabetic foot	30 registered nurses working in medical and endocrinology clinics	A diabetic foot workshop for nurses designed through coordinated sessions covering goal definition, attendee selection, location selection, agenda development and follow‐up planning. Comparator: pre‐training skills and knowledge	The workshop improved nurses' technical and educational skills in diabetic foot assessment and patient follow‐up. The study suggested that structured workshop training may support diabetic foot prevention and potentially reduce ulceration and amputation risk in the long term
Buloala and John 2018 [16]	To assess nurses' knowledge of diabetic foot care before and after interventional training	100 nurses directly involved in the care of patients with diabetes for more than 1 year in hospitals in Rivers State, Nigeria	Training on specialized diabetic foot care using the National Institute for Health and Care Excellence (NICE) diabetic foot care protocol. Comparator: pre‐intervention knowledge levels	Baseline knowledge was low, particularly regarding footwear assessment and assessment of patients' capacity for self‐care. Knowledge of different aspects of diabetic foot care improved significantly after the intervention
Buloala 2019 [17]	To assess nurses' practice of diabetic foot care before and after interventional education	100 nurses directly involved in the care of patients with diabetes for more than 1 year in hospitals in Rivers State, Nigeria	Training on specialized diabetic foot care using the NICE diabetic foot care protocol. Comparator: pre‐intervention practice levels	Practice related to foot inspection, palpation, footwear assessment and assessment of patients' self‐care capacity was low before training. Post‐intervention practice improved across different aspects of diabetic foot care
Fujii and Stolt 2020 [18]	To evaluate a foot‐care educational programme for nurses and care workers at in‐home service providers	110 participants recruited from 21 in‐home service providers	A foot‐care programme delivered as a package with foot‐care tools over 3–5 sessions across 2 months. Comparator: standard care/control group	The intervention group showed significant improvement in foot‐care practice. More than half of participants reported improved learning in several domains. Improved foot‐care practice scores suggested that the educational package was effective despite time constraints in home‐care services
Aalaa et al. 2021 [19]	To compare the educational effects of a diabetic foot care e‐learning course with an interactive workshop of the same content	80 nurses: 40 assigned to a face‐to‐face interactive workshop and 40 assigned to an e‐learning course	Five‐module diabetic foot care training covering diabetic foot definition and ulcer types, foot assessment, ulcer assessment, novel dressing and principles of management. Comparator: interactive workshop versus e‐learning course	Both e‐learning and interactive workshop formats improved nurses' diabetic foot care knowledge. Post‐test learning scores were significantly higher in the interactive workshop group than in the e‐learning group (*p* < 0.01)
Felix et al. 2021 [20]	To compare nurses' knowledge about diabetic foot before and after an educational intervention	53 nurses from the Family Health Strategy in Campina Grande	A 20‐h theoretical‐practical training course on prevention and assessment of diabetic foot. Comparator: pre‐intervention knowledge scores	Nurses' knowledge was deficient before the intervention, with a mean pre‐test score of 23.8 (SD: 12.8). Knowledge increased significantly after the intervention to 41.9 (SD: 9.2; *p* < 0.01), particularly for assessment of plantar protective sensitivity
Ramzan et al. 2022 [21]	To evaluate the effectiveness of an educational programme on nurses' knowledge and practices regarding prevention of diabetic foot ulcers	36 nurses at a tertiary care hospital in Lahore	A 16‐week educational intervention using a booklet and PowerPoint presentation based on guidelines for diabetic foot care. Each session lasted 90 min. Comparator: pre‐intervention knowledge and practice scores	After the intervention, 11.1% of participants had poor knowledge, 36.1% had moderate knowledge and 52.8% had good knowledge. Knowledge and practice scores improved significantly after the intervention (*p* < 0.001)
AbdElshafy et al. 2024 [22]	To assess the effect of a foot care training programme on nurses' knowledge and practices when caring for diabetic geriatric patients	50 nurses providing direct care for geriatric patients	A foot care training programme delivered over 1 month in four sessions: two educational sessions about diabetic foot and two practical training sessions on foot care procedures. Sessions lasted 30–45 min and were delivered in groups of 3–5 nurses. Comparator: pre‐intervention knowledge and practice scores	Knowledge and practice scores improved significantly after training. Place of work predicted baseline knowledge (*p* = 0.009), and nurses with a bachelor's degree showed a greater increase in post‐training knowledge scores (*p* = 0.001)

### 2.11 Data Synthesis

Given the heterogeneity of study designs and outcome measures, no pooled effect size was calculated. Results are presented narratively as reported in the primary studies, including pre‐ and post‐intervention mean scores, proportions and corresponding *p*‐values where available.

## 3 Results

### 3.1 Study Selection

The database search yielded 93 potentially relevant records. After removing 56 duplicate records, 37 unique records remained for screening by title and abstract. During this screening step, fourrecords were excluded because they were clearly unrelated to the review question. The remaining 33 full‐text articles were retrieved and assessed for eligibility against the predefined inclusion and exclusion criteria.

**FIGURE 1:**
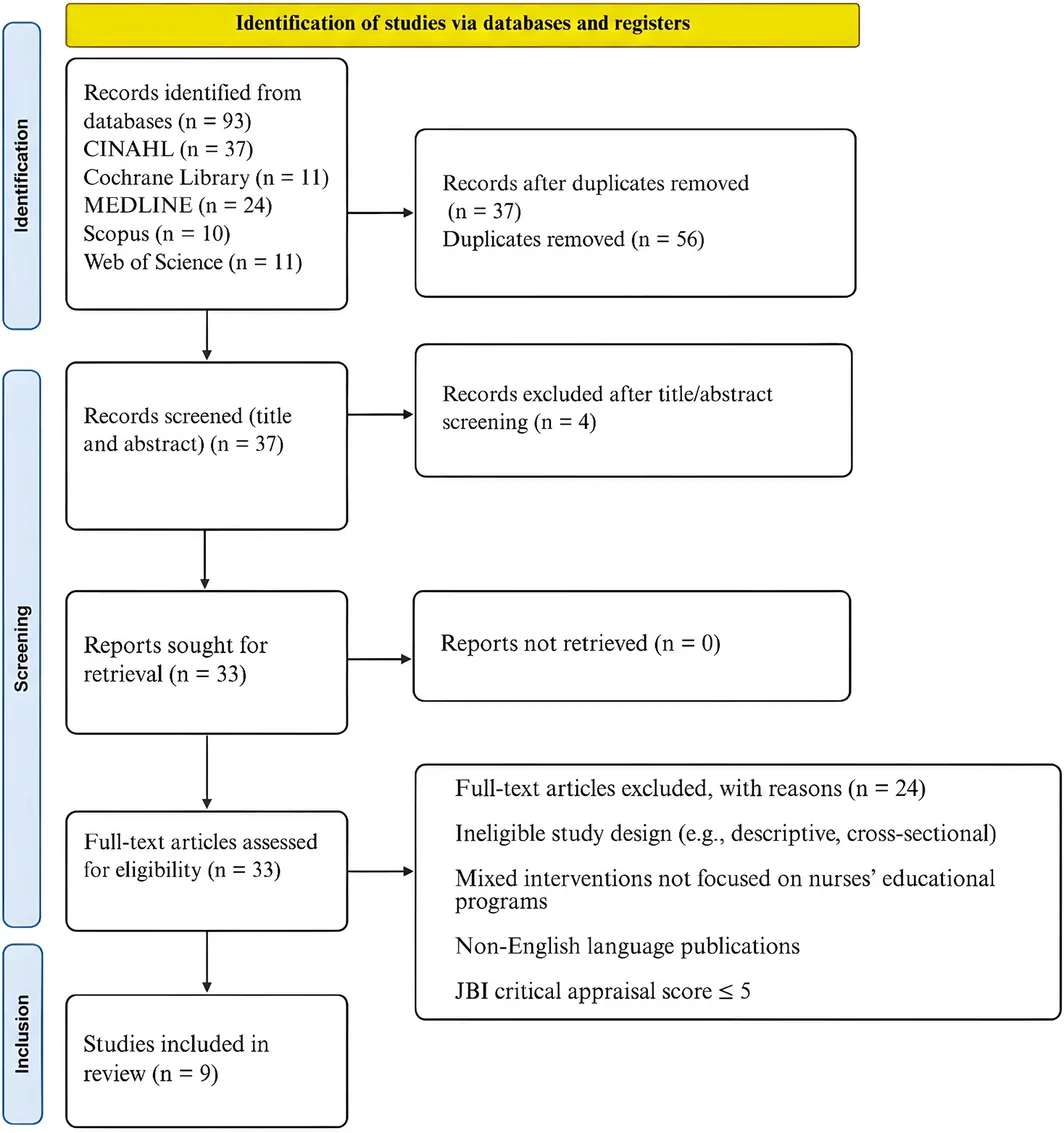
PRISMA 2020 flow diagram of the study selection process.

Of these 33 full‐text articles, 24 were excluded for the following reasons: ineligible study design (e.g., purely descriptive or cross‐sectional studies), mixed interventions that did not focus specifically on nurses' educational programmes for diabetic foot care, non‐English language publications, or a JBI critical appraisal score of 5 or less. A total of nine quasi‐experimental studies met all the inclusion criteria and were included in the final synthesis. The detailed study selection process and the number of records at each stage are presented in the revised PRISMA flow diagram (Figure 1).

### 3.2 Characteristics of Included Studies

Nine quasi‐experimental studies were included in this systematic review [14, 15, 16, 17, 18, 19, 20, 21, 22]. All nine studies were classified as Level II evidence according to the Johns Hopkins Evidence‐Based Practice Model [23]. The sample sizes ranged from 30 to 110 participants, with most studies using a pre–post quasi‐experimental design, while one study used a non‐randomized controlled design with cluster sampling [18], and one study compared two non‐randomized educational delivery formats, namely e‐learning and face‐to‐face interactive workshop training [19]. In several of the included studies, educational programme content was explicitly based on established diabetic foot care guidelines, including those published by the International Working Group on the Diabetic Foot [5] and the template for diabetic foot care [24]. The characteristics and main findings of the included studies are summarized in Table 3.

### 3.3 Narrative Synthesis of Findings

All nine included studies reported statistically significant improvements in nurses' knowledge and/or practice following educational programme delivery, regardless of country, clinical setting or programme format [14, 15, 16, 17, 18, 19, 20, 21, 22]. Knowledge outcomes were assessed in all nine studies, whereas practice‐related outcomes were measured in seven studies. A narrative synthesis was conducted, as heterogeneity in study designs, interventions and outcome measures precluded meta‐analytic pooling.

### 3.4 Certainty of the Evidence

Upon applying the GRADE framework to the synthesized evidence, the overall certainty supporting the impact of educational programmes on nurses' knowledge and clinical practice was rated as ‘very low’ for both outcomes. This rating stems from critical methodological limitations across several domains. First, the risk of bias was judged to be serious; all nine included studies utilized quasi‐experimental designs, predominantly lacking randomization and concurrent control groups, which significantly restricts causal inference. Second, serious inconsistency was noted due to substantial heterogeneity in intervention formats (e.g., workshops, e‐learning, multi‐component packages), programme durations, measurement instruments and follow‐up intervals. Third, imprecision was deemed a serious concern, driven by relatively small sample sizes and a pervasive lack of long‐term follow‐up data beyond the immediate post‐intervention phase.

Consequently, these cumulative limitations yielded an overall ‘very low’ certainty rating, indicating that the true effect of these educational programmes may differ substantially from the estimates reported in the current literature. A synthesis of these GRADE judgements, encompassing the number of studies, participant totals and specific limitations for each outcome, is detailed in the Summary of Findings (Table 4).

**TABLE 4:** Summary of findings (GRADE): Certainty of the evidence for the effects of educational programmes on nurses' knowledge and practice in diabetic foot care.

Outcome	No. of studies (participants)	Study design	Risk of bias	Inconsistency	Indirectness	Imprecision	Overall certainty (GRADE)
Nurses' knowledge of diabetic foot care [14, 15, 16, 17, 18, 19, 20, 21, 22]	589 nurses	Quasi‐experimental, non‐randomized	Serious (no randomization, limited control)	Serious (heterogeneous interventions and measures)	Not serious (population and outcomes directly relevant)	Serious (small samples, short follow‐up)	Very low
Nurses' clinical practice in diabetic foot care [14, 15, 17, 18, 19, 21, 22]	436 nurses	Quasi‐experimental, non‐randomized	Serious	Serious	Not serious	Serious	Very low

### 3.5 Effects on Nurses' Knowledge

Across all studies evaluating knowledge outcomes, educational programmes produced substantial pre‐to‐post improvements. Waheida et al. [14] reported highly significant improvements in nurses' knowledge scores immediately and 1 month after a structured programme combining theoretical instruction with a 60‐s foot screening test (*p* < 0.001), with scores shifting from predominantly poor and fair at baseline to predominantly good at follow‐up. Similarly, Felix et al. [20] found that the mean knowledge score of primary nursing care increased from 23.8 (SD: ±12.8) at baseline to 41.9 (SD: ±9.2) after a 20‐h theoretical and practical training course (*p* < 0.01), with more pronounced gains in areas related to neurological assessment, including the use of monofilaments and tuning forks.

Ramzan et al. [21] showed that all participating nurses had weak knowledge at baseline; after a 16‐week educational intervention, 52.8% achieved good knowledge and 36.1% achieved average knowledge (*p* < 0.001). Buloala and John [16] reported that knowledge of shoe assessment and patient self‐care abilities were significantly lacking before training and improved markedly after a one‐day workshop based on the National Institute for Health and Care Excellence (NICE) protocol (*p* < 0.05). Aalaa et al. [15] also reported similar knowledge gains among nurses who attended a structured 14‐session workshop in Iran, noting improvements in both assessment and patient follow‐up. AbdElshafy et al. [22] found that nurses' knowledge scores improved significantly post‐training across all content areas, with nurses holding a bachelor's degree in nursing demonstrating significantly greater gains than those with lower educational qualifications (*p* = 0.001).

### 3.6 Effects on Nurses' Practice

Studies measuring practice outcomes also reported consistent and significant improvements. Buloala [17] showed that the practice of foot inspection, palpation, footwear assessment and evaluation of patients' capacity for self‐care was unacceptable before training (Likert means < 2.5 for most items) and became acceptable for all assessed items (Likert means ≥ 2.5) following the NICE‐protocol‐based educational intervention. Waheida et al. [14] documented that nurses' practical performance of neurovascular assessment, including standardized use of 10‐g monofilaments, tuning forks and reflex hammers, improved from poor to good categories, with total practical scores of 93.33% and 90% at immediate and 1‐month follow‐up, respectively (*p* < 0.001).

AbdElshafy et al. [22] reported that the proportion of nurses demonstrating good clinical practice increased from 14% at baseline to 100% one month after the training programme, with a statistically significant correlation between total knowledge and practice scores post‐intervention (*p* < 0.001). Fujii and Stolt [18] evaluated a multi‐component foot‐care package delivered across 3–5 sessions over 2 months in home‐care settings; significant improvements were observed in specific practice domains, including skin assessment and referral behaviour, with more than 50% of participants in the intervention group reporting better learning outcomes than controls. Ramzan et al. [21] also reported a significant difference between pre‐ and post‐intervention practice scores among nurses in a tertiary care hospital setting (*p* < 0.001).

### 3.7 Comparison of Delivery Formats

Aalaa et al. [19] provided the only direct comparison of educational delivery formats, evaluating a five‐module e‐learning course against a face‐to‐face interactive workshop of equivalent content among 80 nurses. Both formats produced statistically significant post‐intervention improvements in diabetic foot care knowledge (*p* < 0.01); however, post‐test scores were higher in the interactive workshop group than in the e‐learning group, suggesting that active, participatory formats may be more effective for knowledge acquisition and skill development.

## 4 Discussion

In this systematic review, we evaluated nine quasi‐experimental studies regarding the effectiveness of educational programmes in improving nurses' knowledge and practices in diabetic foot care across different settings and countries. These studies have suggested that structured educational interventions may contribute to nurses' knowledge and practice of diabetic foot prevention and recovery. Although these studies featured different study designs, instruments, durations and clinical settings, the outcomes of each study were statistically significant. At least one of the targeted outcomes (knowledge and/or practice) improved after the intervention, underscoring the potential significance of nursing education in diabetic foot care.

Several studies have shown substantial and clinically significant improvement in nurses' knowledge after relatively short educational interventions. For example, Waheida et al. [14] demonstrated that nurses' knowledge of diabetes and diabetic foot care improved from predominantly poor and moderate levels at baseline to predominantly good levels immediately and 1 month after the implementation of an educational programme (*p* < 0.001). Felix et al. [20] found that primary care nurses' knowledge scores for assessing and preventing diabetic foot increased significantly after a 20‐h theoretical and practical training course, particularly in neurological testing (monofilament and tuning‐fork assessment), which was initially very weak. Ramzan and colleagues [21] also found that all nurses initially had limited knowledge, and more than half had good knowledge after 16 weeks of training and more than a third had intermediate knowledge after that. Together, these results indicate that nurses begin with a low level of knowledge, but structured education appears to be effective in improving nurses' knowledge of diabetic foot risk factors, risk identification and management. The value of structured, multifaceted educational interventions has also been observed in other areas of nursing practice, suggesting that such educational strategies may have broader applicability in enhancing nurses' professional competencies [25].

In addition to knowledge gain, the review highlights that education programmes may lead to better clinical practice of diabetic foot care. Waheida et al. [14] reported significant improvement in nurses' performance on neurovascular assessments (10 g monofilaments, tuning forks and 60‐s diabetic foot screening) with total practical scores improving from poor to good immediately and at 1‐month follow‐up. In an interventional study in Rivers State, Nigeria, Buloala [17] reported that regarding diabetic foot care (foot inspection, palpation, footwear assessment and assessment of patients' self‐care capacity), the practice of specialized diabetic foot care was unacceptable before training (Likert means < 2.5 for most items) but became acceptable (Likert means ≥ 2.5 for all items) following a 1‐day hands‐on workshop.

Likewise, AbdElshafy et al. [22] reported that the proportion of nurses practicing good foot care for diabetic geriatric patients increased from 14% at baseline to 100% 1 month after the training programme, suggesting that well‐designed interventions have the potential to meaningfully change nurses' behaviours at the bedside.

The review also sheds light on different educational formats and their relative effectiveness. Aalaa et al. [19] compared an e‐learning course on diabetic foot care to a face‐to‐face interactive workshop. Both approaches effectively improved nurses' knowledge; however, the interactive workshop yielded higher post‐test scores and larger effect sizes than the e‐learning course, indicating that active, participatory and skills‐based formats may be more effective in improving hands‐on assessment and management skills. Furthermore, the e‐learning approach was found to be more time‐ and cost‐efficient and flexible. Therefore, a hybrid model could offer an accessible and highly effective learning experience.

Another study by Fujii and Stolt [18] further expanded the evidence base by targeting both nurses and care workers in home service providers with a multi‐component programme (PowerPoint, video, booklet, picture cards and on‐site coaching). This intervention resulted in significant improvements in areas of knowledge (nail and skin problems, infection, shoes and sedentary behaviour) and practice (skin assessment and consultation) and suggested that foot care education can be successfully adapted to community and home care contexts beyond hospital settings.

A key observation from these studies is that enhanced knowledge improved clinical practice. AbdElshafy et al. [22] found a statistically significant, moderate correlation between knowledge and overall practice scores after the programme and after 1 month of follow‐up, as nurses' understanding improved and their clinical performance became more in line with recommended standards. This relationship highlights the importance of measuring the quality of knowledge and ensuring that educational programmes are clearly designed to bridge the knowledge‐practice gap through demonstrations, supervised practical training, feedback and time for reflection.

Despite these encouraging findings, important gaps and challenges remain in the available evidence. Many nurses in various countries reported insufficient or no formal training in diabetic foot care, especially in primary healthcare centres and general hospitals. This contributes to a lack of basic knowledge and suboptimal practice in neurological assessment, monofilament testing, shoe evaluation and systematic foot examination. Several studies indicate a lack of essential equipment (such as 10‐g monofilaments, tuning forks and reflex hammers) in primary healthcare services.

Consequently, nurses are forced to use inappropriate tools or omit parts of the examination, even after receiving training. This structural limitation may hinder the translation of acquired knowledge into clinical practice, thereby reducing opportunities for positive change. Moreover, although most interventions have improved knowledge and practice in the short run, few studies have assessed long‐term skill retention or the impact of nurse training on patient outcomes such as ulcer incidence, amputation rates or hospital admissions.

This review builds on the existing literature by specifically investigating nurse education programmes and nurse‐led foot care for diabetic foot prevention, rather than focusing solely on patient education. It reaffirms that nurses are integral members of multidisciplinary diabetic foot care teams and play central roles in assessment, risk stratification and patient education, all of which are relevant to clinical outcomes. By synthesizing evidence from the Middle East, South Asia, South America, Africa and East Asia, this review shows that the need for and the benefits of nurse education in diabetic foot care are universal and not specific to any one healthcare system.

At the same time, differences in study designs, outcome measures and intervention content make it difficult to compare effect sizes and determine the optimal dose and format of instruction. Most included studies used quasi‐experimental pre‐ and post‐test designs without randomization or control groups, increasing the risk of selection bias and limiting causal inference. Only a few studies have more rigorous designs (i.e., non‐randomized controlled trials with cluster sampling) or detailed psychometric properties for their instruments. Future research should address these methodological shortcomings and standardize outcome measures in order to provide a more robust evidence base.

### 4.1 Implications for Practice and Future Research

The findings of this review have potential implications for nursing practice, education and health policy. First, as noted in the review, nursing education in diabetic foot care represents a vital element of continuing professional development in all settings where people with diabetes receive care (hospitals, outpatient clinics, primary care centres and home care). Training programmes on risk assessment, neurovascular assessment, the correct use of monofilament and tuning forks, footwear assessment and patient counselling could be effectively integrated into institutional curricula. Evidence suggests that interactive workshops and practical classes enhance skill acquisition in clinical practice more effectively than traditional lectures or online teaching.

Second, education programmes should be tailored to nurses' specific needs. In settings where nurses have limited prior experience in diabetic foot care, training may initially focus on basic definitions, pathophysiology and risk factors before progressing to more complex topics. In specialized diabetes clinics or geriatric units, however, the focus may instead be placed on complex cases and high‐risk patients. Geriatric nurses, community nurses and care workers should also be included in foot care training, particularly given the increasing provision of diabetes care in community and home‐care settings.

Third, the reviewed studies suggest that educational programmes alone are insufficient; adequate resources and organizational support are also required. Health care providers and policy makers should ensure that diabetic foot assessment tools (10‐g monofilaments, tuning forks, portable Doppler devices and other footwear assessment tools) are available in primary care clinics and hospitals. Clinical protocols and pathways for diabetic foot screening and referral should be established or updated in line with international guidelines. Nurses must be given formal authority and encouraged to carry out regular foot examinations during consultations and hospital admissions. Managers should also allocate sufficient time for nurses to attend training courses and perform systematic foot assessments.

With regard to future research, we require more thorough and well‐designed trials to evaluate different teaching strategies to enhance nurses' skills in diabetic foot care. Randomized or cluster‐randomized controlled trials comparing independent e‐learning, face‐to‐face workshops, blended learning, mentoring models and clinically integrated teaching would better identify the best and most sustainable teaching approaches to improve practice. Longitudinal studies assessing the sustainability of training effects over 6–12 months and beyond are also required. Most existing studies only measure outcomes after or soon after the intervention. Furthermore, future research needs to be more focused on patient outcomes (new or recurrent foot ulcers, amputation rates, hospital admissions, quality of life) to identify whether nursing education programmes are translating into real‐life benefits for people with diabetes.

Finally, developing standardized measures of nurses' knowledge and practice in diabetic foot care would improve comparability across different settings. Researchers should also consider the contextual and organizational factors (workload, staffing levels, equipment availability and leadership support) that may help or hinder the application of learning gains in routine practice. Mixed‐methods approaches incorporating qualitative components to explore nurses' experiences, barriers and enabling factors may help inform the design of more effective and acceptable educational interventions.

### 4.2 Limitations

This systematic review has several limitations that should be considered when interpreting the findings. First, the majority of studies employed quasi‐experimental pre‐ and post‐intervention designs without randomization (and, in some cases, lacked a control group). Consequently, improvements in knowledge and practice may be attributed to maturation, repeated testing or other uncontrolled effects rather than solely the educational interventions. Only a few studies employed more robust designs, and none were randomized controlled trials, which limits the strength of causal inference.

Second, there was considerable heterogeneity in intervention content, duration, number of sessions, teaching methods, participant populations and outcome measures. This variation prevents a systematic meta‐analysis and makes direct comparisons of effect sizes between studies difficult. Additionally, some studies used locally developed instruments with limited evidence of validity and reliability, potentially introducing measurement bias.

Third, most studies measured outcomes only in the short term, typically immediately after completion of the intervention or within 1 month. Data on long‐term retention of knowledge and skills were very limited, and none of the included studies indicated whether the acquired nursing skills were maintained for months or years. Furthermore, most studies only examined nurse‐level outcomes (knowledge and practice) and did not assess patient‐level outcomes like ulcer incidence, amputation rates or hospital admissions, so it is difficult to assess the clinical significance of educational programmes.

Fourth, this review was restricted to English‐language publications within a specific time frame, potentially introducing linguistic and publication bias. The relevant papers in other languages or in grey literature (dissertations, conference proceedings and institutional reports) may have been missed. Some of the studies included were small and were done in isolated centres, which restricts the generalizability of their findings to other healthcare settings and systems. Finally, despite the use of predefined eligibility criteria and a standardized appraisal tool, some degree of subjectivity in study selection and quality assessment remains. Moreover, variability in the reporting quality of primary studies limited the available information regarding intervention content, implementation context and delivery methods. Despite these limitations, the consistent direction of impact across various settings and designs supports the belief that nurse education programmes can be beneficial for the knowledge and practice of diabetic foot care.

## 5 Conclusion

Findings from this systematic review suggest that educational programmes may be associated with improvements in nurses' knowledge and, to a lesser extent, clinical practice related to diabetic foot care across diverse healthcare settings. All nine included studies reported statistically significant pre‐to‐post improvement in at least one targeted outcome; however, given that all studies employed quasi‐experimental designs, that most lacked randomization or a concurrent control group and that several had relatively small sample sizes, the overall certainty of this evidence, as assessed using GRADE, is very low. Consequently, these findings should be interpreted as suggestive rather than conclusive, and causal claims regarding the effectiveness of educational interventions cannot be firmly established from the current evidence base. Structured, interactive and hands‐on educational approaches appear promising relative to passive or purely online formats, but this observation warrants confirmation through more rigorous, adequately powered randomized or cluster‐randomized controlled trials before firm practice recommendations can be made. Nursing educators and policymakers may consider integrating competency‐based diabetic foot care training into professional development programmes as a reasonable interim strategy, while prioritizing higher‐quality research that examines long‐term skill retention and patient‐level outcomes such as ulcer incidence and amputation rates.

## Author Contributions

**Omar Alqaisi:** methodology, writing – review and editing, formal analysis, investigation. **Heyam Al‐Aaraj:** writing – original draft, conceptualization, investigation. **Mohammad Al‐Me'ani:** conceptualization, methodology, validation, writing – original draft, formal analysis. **Mohammed Dibas:** methodology, software, writing – review and editing, project administration. **Yousef Rubbai:** writing – original draft, conceptualization, investigation.

## Funding

The authors have nothing to report.

## Ethics Statement

This systematic review is based exclusively on previously published studies. No primary data were collected from human participants; therefore, ethical approval were not required.

## Consent

The authors have nothing to report.

## Conflicts of Interest

The authors declare no conflicts of interest.

## Supporting information


**Appendix S1:** Full database search strategies.

## Data Availability

Data sharing is not applicable to this article as no datasets were generated or analysed during the current study.
