# Supplementary material for: Effectiveness of Educational Programmes on Knowledge and Practice Related to Diabetic Foot Care Among Nurses: A Systematic Review
Source: Endocrinol Diabetes Metab. 2026 Aug 27;9(5):e70324. doi: 10.1002/edm2.70324 (PMC13521569; doi:10.1002/edm2.70324)
Supplement: Supplementary file 1 [file EDM2-9-e70324-s001.docx]

Appendix 1: Full Database Search Strategies

BLOCK 1 POPULATION (P): Nurses

| **Database** | **Search String** |
| --- | --- |
| MEDLINE (PubMed) | "Nurs*"[Title/Abstract] OR "Nursing Staff"[MeSH Terms] OR "Nurses"[MeSH Terms] OR "Registered Nurse*"[Title/Abstract] OR "Nursing Practice"[MeSH Terms] OR "Clinical Nurs*"[Title/Abstract] OR "Community Nurs*"[Title/Abstract] |
| CINAHL (EBSCOhost) | (TI Nurs* OR AB Nurs*) OR (MH "Nurses") OR (MH "Nursing Practice") OR (TI "Registered Nurse*" OR AB "Registered Nurse*") OR (TI "Community Nurse*" OR AB "Community Nurse*") |
| Scopus | TITLE-ABS-KEY(Nurs* OR "Registered Nurse*" OR "Nursing Staff" OR "Nursing Practice" OR "Clinical Nurse*" OR "Primary Care Nurse*") |
| Web of Science | TS=(Nurs* OR "Registered Nurse*" OR "Nursing Staff" OR "Nursing Practice" OR "Clinical Nurs*") |
| Cochrane Library | "Nurs*" OR "Nursing Staff" OR "Registered Nurse*" OR "Nursing Practice" (Title, Abstract, Keywords) |

BLOCK 2 INTERVENTION (I): Educational Program / Training

| **Database** | **Search String** |
| --- | --- |
| MEDLINE (PubMed) | "Educational Program*"[Title/Abstract] OR "Educational Intervention*"[Title/Abstract] OR "Training Program*"[Title/Abstract] OR "Workshop*"[Title/Abstract] OR "E-learning"[Title/Abstract] OR "In-service Training"[Title/Abstract] OR "Continuing Nursing Education"[MeSH Terms] OR "Staff Development"[MeSH Terms] |
| CINAHL (EBSCOhost) | (TI "Educational Program*" OR AB "Educational Program*") OR (TI "Training Program*" OR AB "Training Program*") OR (TI "Educational Intervention*" OR AB "Educational Intervention*") OR (TI Workshop* OR AB Workshop*) OR (MH "Inservice Training") OR (MH "Staff Development") |
| Scopus | TITLE-ABS-KEY("Educational Program*" OR "Educational Intervention*" OR "Training Program*" OR "Workshop*" OR "E-learning" OR "Staff Development" OR "In-service Training" OR "Continuing Education") |
| Web of Science | TS=("Educational Program*" OR "Educational Intervention*" OR "Training Program*" OR "Workshop*" OR "E-learning" OR "Staff Development" OR "In-service Training" OR "Continuing Education") |
| Cochrane Library | "Educational Program*" OR "Educational Intervention*" OR "Training Program*" OR "Workshop*" OR "E-learning" OR "Staff Development" (Title, Abstract, Keywords) |

BLOCK 3 CONDITION (C): Diabetic Foot Care

| **Database** | **Search String** |
| --- | --- |
| MEDLINE (PubMed) | "Diabetic Foot"[MeSH Terms] OR "Diabetic Foot Care"[Title/Abstract] OR "Diabetic Foot Ulcer*"[Title/Abstract] OR "Foot Care"[Title/Abstract] OR "Foot Ulcer"[MeSH Terms] OR "Foot Assessment"[Title/Abstract] OR "Diabetic Foot Prevention"[Title/Abstract] |
| CINAHL (EBSCOhost) | (TI "Diabetic Foot*" OR AB "Diabetic Foot*") OR (TI "Foot Care" OR AB "Foot Care") OR (TI "Diabetic Foot Ulcer*" OR AB "Diabetic Foot Ulcer*") OR (MH "Diabetic Foot") OR (TI "Foot Assessment" OR AB "Foot Assessment") |
| Scopus | TITLE-ABS-KEY("Diabetic Foot" OR "Diabetic Foot Care" OR "Diabetic Foot Ulcer*" OR "Foot Care" OR "Foot Ulcer*" OR "Foot Assessment" OR "Diabetic Foot Management") |
| Web of Science | TS=("Diabetic Foot" OR "Diabetic Foot Care" OR "Diabetic Foot Ulcer*" OR "Foot Care" OR "Foot Ulcer*" OR "Foot Assessment" OR "Diabetic Foot Management") |
| Cochrane Library | "Diabetic Foot" OR "Diabetic Foot Care" OR "Diabetic Foot Ulcer*" OR "Foot Care" OR "Foot Assessment" (Title, Abstract, Keywords) |

BLOCK 4 OUTCOME (O): Knowledge and Practice

| **Database** | **Search String** |
| --- | --- |
| MEDLINE (PubMed) | "Knowledge"[Title/Abstract] OR "Practice"[Title/Abstract] OR "Clinical Competenc*"[Title/Abstract] OR "Skills"[Title/Abstract] OR "Awareness"[Title/Abstract] OR "Performance"[Title/Abstract] |
| CINAHL (EBSCOhost) | (TI Knowledge OR AB Knowledge) OR (TI Practice OR AB Practice) OR (TI "Clinical Skills" OR AB "Clinical Skills") OR (TI "Clinical Competenc*" OR AB "Clinical Competenc*") OR (TI Performance OR AB Performance) |
| Scopus | TITLE-ABS-KEY(Knowledge OR Practice OR "Clinical Competenc*" OR Skills OR Awareness OR Performance OR "Clinical Skills") |
| Web of Science | TS=(Knowledge OR Practice OR "Clinical Competenc*" OR Skills OR Awareness OR Performance OR "Clinical Skills") |
| Cochrane Library | Knowledge OR Practice OR "Clinical Skills" OR Competenc* OR Performance (Title, Abstract, Keywords) |

FINAL COMBINED SEARCH All Blocks (P AND I AND C AND O)

| **Database** | **Final Combined String** | **Filters Applied** |
| --- | --- | --- |
| MEDLINE (PubMed) | (#1) AND (#2) AND (#3) AND (#4) | Year: 2015–2024 \| Language: English \| Full text |
| CINAHL (EBSCOhost) | (S1) AND (S2) AND (S3) AND (S4) | Published: 2015–2024 \| Language: English \| Full text |
| Scopus | (#1) AND (#2) AND (#3) AND (#4) | Year: 2015–2024 \| Language: English \| Article type: Article |
| Web of Science | (#1) AND (#2) AND (#3) AND (#4) | Year: 2015–2024 \| Language: English \| Article type: Article |
| Cochrane Library | (#1) AND (#2) AND (#3) | Year: 2015–2024 \| Language: English |

In the Cochrane Library, Block 4 (Outcome) was omitted from the final combination to avoid over-restriction, as Cochrane records primarily comprise systematic reviews and RCTs with well-defined outcomes.

Total records retrieved: 93, After deduplication: 56, After title/abstract screening: 33, Final included: 9.
